# Marine Sediment Mixed With Activated Carbon Allows Electricity Production and Storage From Internal and External Energy Sources: A New Rechargeable Bio-Battery With Bi-Directional Electron Transfer Properties

**DOI:** 10.3389/fmicb.2019.00934

**Published:** 2019-05-14

**Authors:** Emilius Sudirjo, Cees J. N. Buisman, David P. B. T. B. Strik

**Affiliations:** ^1^Government of Landak Regency, West Kalimantan, Indonesia; ^2^Environmental Technology, Wageningen University & Research, Wageningen, Netherlands

**Keywords:** activated carbon, capacitance, bio-battery, bio anode, marine sediment, charging, discharging, energy storage

## Abstract

Marine sediment has a great potential to generate electricity with a bioelectrochemical system (BES) like the microbial fuel cell (MFC). In this study, we investigated the potential of marine sediment and activated carbon (AC) to generate and store electricity. Both internal and external energy supply was validated for storage behavior. Four types of anode electrode compositions were investigated. Two types were mixtures of different volumes of AC and Dutch Eastern Scheldt marine sediment (67% AC and 33% AC) and the others two were 100% AC or 100% marine sediment based. Each composition was duplicated. Operating these BES’s under MFC mode with solely marine sediment as the anode electron donor resulted in the creation of a bio-battery. The recharge time of such bio-battery does depend on the fuel content and its usage. The results show that by usage of marine sediment and AC electricity was generated and stored. The 100% AC and the 67% AC mixed with marine sediment electrode were over long term potentiostatic controlled at -100 mV vs. Ag/AgCl which resulted in a cathodic current and an applied voltage. After switching back to the MFC operation mode at 1000 Ω external load, the electrode turned into an anode and electricity was generated. This supports the hypothesis that external supply electrical energy was recovered via bi-directional electron transfer. With open cell voltage experiments these AC marine bioanodes showed internal supplied electric charge storage up to 100 mC at short self-charging times (10 and 60 s) and up to 2.4°C (3,666 C/m^3^ anode) at long charging time (1 h). Using a hypothetical cell voltage of 0.2 V, this value represents an internal electrical storage density of 0.3 mWh/kg AC marine anode. Furthermore it was remarkable that the BES with 100% marine sediment based electrode also acted like a capacitor similar to the charge storage behaviors of the AC based bioanodes with a maximum volumetric storage of 1,373 C/m^3^ anode. These insights give opportunities to apply such BES systems as e.g., *ex situ* bio-battery to store and use electricity for off-grid purpose in remote areas.

## Introduction

Sediment microbial fuel cells (SMFC) are among the most studied bioelectrochemical systems (BESs) which attracts attention from many researchers because of its ability to generate power and provide bioremediation (Abbas et al., 2017). In a SMFC the anode is for example placed into the anaerobic sediment and the cathode is placed on the upper position of the aerobic water layer (Reimers et al., 2001; Logan and Regan, 2006; Lowy et al., 2006). In this paper, we will demonstrate the use of the SMFC for energy storage purposes.

Sediment contains not only organic matters (Mathuriya and Yakhmi, 2016) but also abundant electrochemically active bacteria (EAB) communities to generate electricity in the SMFC (Logan and Regan, 2006). With these properties, the SMFC can be implemented as *in situ* renewable electricity source (Tender et al., 2002). The SMFC was tested for applications as an *in situ* renewable power source for long term monitoring instruments like the oceanographic instrument, meteorological buoy, acoustic modem, telecommunication system, remote sensor, submersible ultrasonic receiver, turbidity meter, acoustic receiver or wireless temperature probe (Reimers et al., 2001; Zabihallahpoor et al., 2015).

In theory, the microbial fuel cell (MFC) can continuously generate electricity as long as there is enough substrate to be utilized by EAB (Logan et al., 2006). Apparently for the *in situ* SMFC system, the substrate availability will not be a direct limiting factor to generate electricity at long terms because enormous amount of organic matter is present and supplied to the sediment (Middelburg et al., 1996; de Haas et al., 1997; Seiter et al., 2004).

The sediment organic matter is a primary energy source of the SMFC to produce power (Reimers et al., 2001). Marine or sea sediment is well known to be rich with organic carbon as a result of photosynthetic fixation of inorganic carbon by terrestrial and marine phytoplankton (Seiter et al., 2004). In coastal areas, these marine sediments can be inhabited by higher plants like *Spartina anglica*. The organic carbon in sediments can be measured as total organic carbon (TOC). In a low salt marsh estuarine intertidal sediment, which is dominated by *Spartina anglica* vegetation, the TOC at depth 0–0.2 m is about 2% (Van de Broek et al., 2016). This TOC gives a kind of maximum available fuel content of a SMFC.

There are two common methods to utilize MFC power for relatively high voltage applications, either using a DC-DC converter or using a capacitor (Kim et al., 2011). A DC-DC converter allows us to continuously power low power consuming devices (Dewan et al., 2009). For example an SMFC was successfully powering a wireless telecommunication system by integrating a SMFC system and DC-DC converter (Thomas et al., 2013). A capacitor makes it also possible to intermittently powering high power consuming devices since electric charge is stored over time and released once needed (Dewan et al., 2009).

In addition to substrate availability, also the electrode materials play an important factor for an MFC. The anode electrode is a structure which serves as an electron acceptor for the EAB. It is important to find an inexpensive and suitable material combining conductivity and high surface area with three dimensional structures (Logan et al., 2006; Kumar et al., 2013; Xie et al., 2015). Moreover, for integrating MFC technology with biomass production by planting plants, the anode material should be able to support plant’s roots and be able to restore the electrical connectivity in the anode after disturbance (Arends et al., 2012).

Among possible anode materials, activated carbon (AC) granules seems to be a promising. Despite that (some) AC this apparent less conductive compare to graphite granule (Huggins et al., 2014), its large surface area and porous structure (Zhang and Zhao, 2009) is suited for EAB growth. Several researches have shown that bacteria are able to grow on the AC and are forming biofilms (Kalathil et al., 2011; Manickam et al., 2013; Borsje et al., 2016). This biofilm has also shown a capability to store charge in the form of electrons in multi-heme c-type cytochromes (Liu and Bond, 2012; Malvankar et al., 2012). In addition to its prospective to be a bioanode, AC has also a capacitive electron storage capability. Recent research on a single AC granule has shown that the AC can store electric charge (Borsje et al., 2016). This capacitive property is opening possibilities to store *in situ* generated electricity within the MFC (Deeke et al., 2012). In addition, the system can be considered as a bio-battery.

A bio-battery is an energy storing system based on the redox reaction of organic compounds with the help of enzymes or bacteria. A bio-battery also has an anode, cathode, separator and electrolyte. In the anode, electrons and hydrogen ions are generated from oxidation reaction of sugar type organic compound, i.e., glucose. The hydrogen ions migrate to the cathode through a separator, and, together with electrons that pass through the outer circuit, they reduce oxygen into water (Kannan et al., 2009).

Considering the AC’s properties mentioned above and the benefit of sediment it seems possible to integrate both of them in a MFC based bio-battery. Therefore, the objective of this study was to investigate the abilities of marine sediment and activated carbon to store and generate electricity in a bio-battery. This work allowed the development of a new kind of bio-battery with bi-directional electron transfer properties. Both external and internal supplied energy i.e., generated electricity could be stored at different time domains. To understand the behavior of the bio-battery, several experiments were conducted to clarify: (i) the role of the sediment in providing fuel; (ii) the role of AC in supporting bi-directional electron transport behavior; and (iii) the role of sediment and AC on *in situ* charge storage behavior.

## Materials and Methods

### Experimental Setup

**FIGURE 1:**
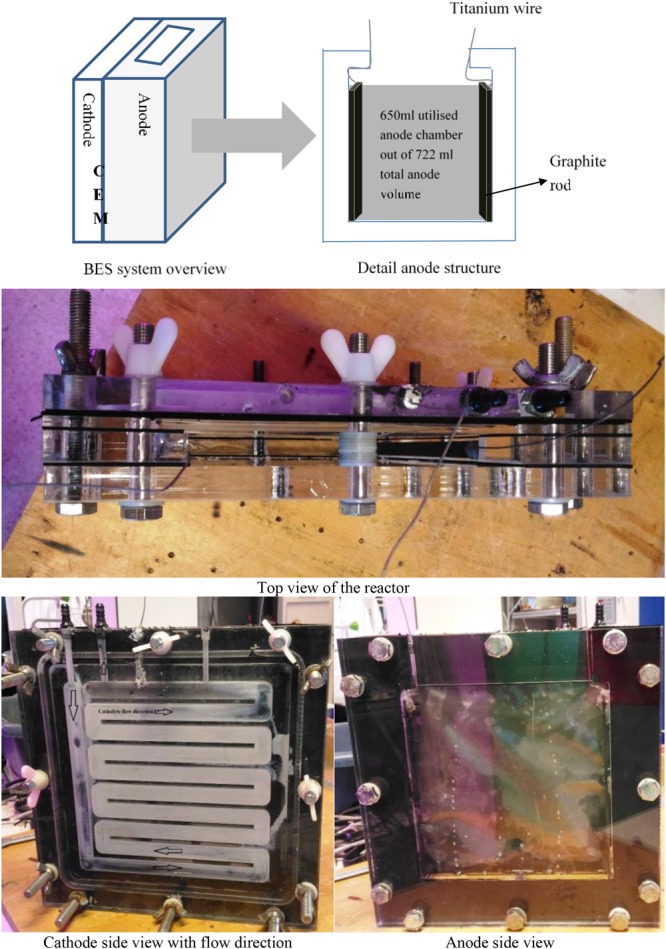
Bioelectrochemical system (BES) reactor schemes and pictures from different positions.

Eight identical flat plates BES reactors made of acrylic glass were utilized for this experiment similar to Wetser et al. (2015b). The vertically placed reactors had two compartments that either functioned as an anode or a cathode. Both compartments were separated with a cation exchange membrane (CEM) fumasep FKD-PK-75 PEEK-reinforces, 75 μm. The anode compartment had a total volume of 722 ml (19 cm × 19 cm × 2 cm) but only 650 ml were filled with anode material. The anode compartment had an open space on the top (19 cm × 2 cm). Two graphite rods (18 cm × 1 cm × 0.2 cm) were used as current collector. The current collectors was connected with titanium wire (1 mm diameter) and glued in both side of the anode chamber (Figure 1). Stages of the BESs reactor preparation was presented in Supplementary Figure 4.

In the cathode i.e., counter electrode compartment (22 cm × 22 cm × 1 cm; with a winding channel for catholyte flow), graphite felt 22 cm × 22 cm (3 mm thickness, Grade WDF, National specialty product carbon and Graphite Felt, Taiwan) was used as an electrode. This electrode was woven with a titanium wire as a current collector. Nitrate-less, sulfate-less, ammonium bicarbonate-rich plant growth medium was utilized as catholyte (Helder et al., 2012). The catholyte was aerated with ambient air using an aquarium pump and recirculated into the cathode chamber in a close cycle via a 1 L bottle with a pump (Watson-Marlow 505S, Rotterdam, Netherlands at 30 rpm). Total catholyte volume in the close cycle was maintained at 1 L. Both anode and cathode potential were measured and reported against 3M KCl Ag/AgCl reference electrode.

This study utilized four different anode compositions which served as the electrode. The four anode compositions were two of mixing different volumes of AC (PK 1-3 Cabot Norit Nederland BV, with apparent density of 290 g/L) and mixed with marine sediment (67 and 33% AC), 100% AC and 100% marine sediment (Table 1). The anode was further mixed with Nitrate-less, sulfate-less, ammonium-bicarbonate-rich plant growth medium (Helder et al., 2012) that was utilized as the anolyte (the exact composition is also given in the Supplementary Table 2). Each composition was duplicated. The marine sediment (with density of 1.58 g/mL) was collected from tidal area of the Eastern Scheldt of the North Sea at Krabbendijke, Zeeland Province, Netherlands (51.446710N, 4.093149E).

**Table 1:** Anode composition.

BES	Volume		Composition		Composition	
	Percentage		(mL)		(gr)	
	AC	Marine sediment	AC	Marine sediment	AC	Marine Sediment
1 and 2	100%	0%	650	0	188.5	0
3 and 4	0%	100%	0	650	0	1027
5 and 6	67%	33%	435.5	214.5	126.3	338.9
7 and 8	33%	67%	214.5	435.5	62.2	688.1

### Operational of the Reactors

All BES reactors were operated for 156 days. Within these 156 days two different experiments were conducted, which were the power generation experiment (day 1–72 and day 96–118) and the electricity storage experiment (day 72–96 and day 118–156). During the power generation experiment (MFC mode) two types of controls were alternately applied. First, an external load control in which the anode and the cathode were connected with 1000 Ω external load (day 1–5, day 14–44, day 56–72, day 96–118). Secondly, a potentiostat control (day 5–14, day 44–56) in which the anode potential was maintained at -100 mV vs. Ag/AgCl (Transients, Chronoamperometry) with a potentiostat (Ivium Technologies, Eindhoven, Netherlands). The anodes were controlled with a three electrode setup in which the anode was the working electrode, the cathode as the counter electrode and a reference electrode (Ag/AgCl type No: QM710X from QIS, Oosterhout, Netherlands) in the anode as the reference electrode. On day 105, 2 g/L of acetate in form of sodium acetate (NaAc) was added into each anode of the BESs and another 2 g/L NaAc was added to the anode of BES 1 and 2 on day 117 after sampling. The system was operated in the light and dark ratio of 14:10 h within a climate chamber (Microclima 1750, Snijders Scientific, Tilburg, Netherlands) at 20°C and humidity of 70% similar to Wetser et al. (2015b).

During the electricity storage experiments, the reactors were only controlled with a potentiostat (Transients, mixed Mode). The experiment was executed by self-charging at open circuit and followed by discharging the BESs at 65 mV anode potential. I.e., mode 1 (charging period) was set in open cell and mode 2 (discharge period) was set to a fixed potential (65 mV). Each set of electricity storage experiments was performed for 40 times. Stored charge of the final 10 cycles was calculated as explained by Deeke et al. (2012), which is summarized as following Eq1:

(1) $$\begin{array}{c} Q_{s}{=Q}_{m}{– Q}_{cont,d}\; \end{array}$$

where *Q*_s_ is the stored charge (C); *Q*_m_ is the measured charge (C) during discharge period which was logged with IviumSoft; and *Q*_cont,d_ is the expected charge (C) at a steady-state current (A). The expected charge is a product of steady-state current (A) and time (t) during the discharge period. The steady-state current in this calculation was the average current of the last minute of each cycle.

Charge recovery and energy recovery was calculated within the first power generation experiment period from day 44 until 72. The charge recovery was calculated based on the Coulombs supplied current during the potentiostatic control (day 44 until day 55) versus Coulombs extracted during the external load control (day 56 until day 72) as given by Eq(2).

(2) $$\begin{array}{c} Charging\;recovery=\frac{\Sigma Q_{discharging}}{\Sigma Q_{charging}} \end{array}$$

(3) $$\begin{array}{c} Q_{charging}=I_{avg.charging}\times t_{charging} \end{array}$$

where *Q_charging_* is charge supplied during a potentiostatic control; *I_avg.charging_* is average current during potentiostatic control; and *t_charging_* is duration of the charging time. *Q_charging_* was calculated on a daily basis for day 44 until day 55 and the result was summarized as Σ*Q*_charging_.

(4) $$\begin{array}{c} Q_{discharging}=I_{avg.discharging}\times t_{charging} \end{array}$$

where *Q_discharging_* is charge extracted during the external control; *I_avg.dischrging_* is average current during the external load control; and *t_discharging_* is duration of the discharging time. *Q_discharging_* was also calculated on a daily basis for day 56 until day 72 and the result was summarized as Σ*Q_discharging_*.

The energy recovery was a ratio between total output energy during the external load control (day 56 until day 72) and total input energy during the potentiostatic control (day 44 until day 55).

(5) $$\begin{array}{c} Energy\;recovery=\frac{E_{out}}{E_{in}} \end{array}$$

Both output energy and input energy were calculated on a daily basis according to following Eqs 6, 7:

(6) $$\begin{array}{c} E_{input}=E_{applied}\times Q_{charging} \end{array}$$

(7) $$\begin{array}{c} E_{out}=E_{cell}\times Q_{discharging} \end{array}$$

where *E_input_* is input energy (J); *E_output_* is output energy (J); *E_applied_* is applied cell potential during charging (-100 mV) and *E_cell_* is average obtained cell voltage during discharging at 1000 Ω.

### Measurement and Analysis

From day 21 until day 156, anode potentials, cathode potentials, membrane potentials and cell potentials were logged with a field point (National Instruments FP-2000; FP-AI-112) similar to Helder et al. (2012) and Wetser et al. (2015b). Prior to mentioned period, the anode potentials, the cathode potentials and the cell potential were manually measured with a multimeter. Apart from data logger, during potentiostat control generated current was logged with IviumSoft of Ivium Technologies connected to a lab PC.

Every 1 or 2 weeks, liquid samples were taken from the anode and the cathode. Anolyte samples were taken using filtered syringe and catholyte samples were taken from cathode outlet before entering recirculating bottle. Samples were stored in -20°C for further analysis. Conductivity and pH were measured right after sample collections. Conductivity was measured using HQ440d multi pH/LDO/conductivity meter HACH and pH was measured using a PHM210 standard pH meter, MeterLab Radiometer analytical.

Acetate concentrations were determined by gas chromatography (Agilent 7890B, United States) as described earlier (Jourdin et al., 2018). An HP-FFAP Column was used (25 m × 0.32 mm × 0.50 μm). The detector (FID) and injection temperatures were 240 and 250°C, respectively. The oven temperature was 60°C for 3 min, 21°C min^-1^ up to 140°C, 8°C min^-1^ up to 150°C and constant for 1.5 min, 120°C min^-1^ up to 200°C and constant for 1.25 min, and finally 120°C min^-1^ up to 240°C and constant for 3.5 min. Helium was used as carrier gas at a flow of 1.25 mL min^-1^ for the first 3.5 min and 2 mL min^-1^ until the end of the run. 1 μL of sample was injected in the column. Acetate concentration result can be found in the Supplementary Figure 3.

## Results and Discussion

### Dutch Eastern Scheldt Marine Sediment Is a Suitable Fuel to Generate Electricity With a Bio-Battery

**FIGURE 2:**
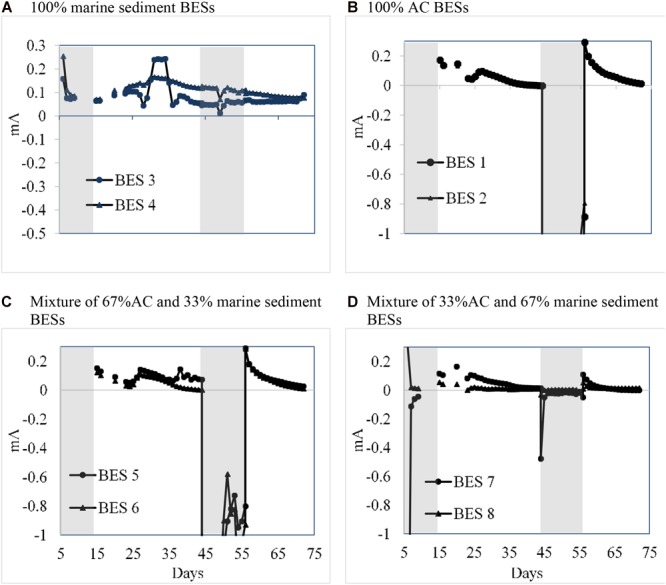
Average daily current generation on different control modes for **(A)** 100% sediments BES 3 and 4, **(B)** 100% AC BES 1 and 2, **(C)** mixing 67% AC BES 5 and 6, **(D)** mixing 33% AC BES 7 and 8. Shaded area: Potentiostatic control mode; non-shaded area: external load control.

The Eastern Scheldt marine was a suitable fuel to generate electricity with the BES. The designed system acted as a bio-battery. During the first 72 days operation of the two marine sediment BESs (BESs 3 and 4; i.e., withoutAC), electricity was continuously generated during MFC operation mode (Figure 2A). In this period no additional substrate (acetate) was added. On average both BESs generated 0.1 ± 0.09 mA, which correlates with current density 26.3 mA/m^2^ land use area (154 mA/m^3^ anode volume) and a consequent power density of 2.63 mW/m^2^ land (15.4 mW/m^3^ anode volume). This result is lower than generated power with a graphite rod anode and intertidal sediment, which was 19.6 mW/m^2^ projected land use (Sacco et al., 2012). A 50 mA/m^2^ projected land use (20 mW/m^2^) was reached with a 3D carbon cloth marine sediment anode while up to 100 mA/m^2^ projected land use (55 mW/m^2^) was reached with a carbon sponge marine sediment anode (Scott et al., 2008). This result is also lower than a planted (*Spartina anglica*) marine sediment MFC which reached a 18 mW/m^2^ (83 mA/m^2^) plant growth area (Wetser et al., 2015a). However, later on in the experiment the performance of these BESs were improved with a current density in range of 41–71 mA/m^2^ possibly due to a more mature bioanode development (see Supplementary Table 3 for a complete performance set of all operated BESs).

In the studied 100% marine sediment BESs, just two small solid graphite sticks were used as both current collector and as anode electrode. These current collectors (0.00252 m^2^ each) were vertically placed at both sides of the anode compartment with 18 cm distance (Figure 1). The open space between the current collectors was filled with marine sediment. The electricity was generated within the bioanode while electrons were collected via the current collector. It is known that marine sediment can have apparent conductive properties which can also support transfer of generated electrons and/or ions from the bacteria in the sediment to the current collector (Roden et al., 2010; Malvankar et al., 2014). Possibly that this phenomena was also apparent within this studied BES, however this was not further validated.

**FIGURE 3:**
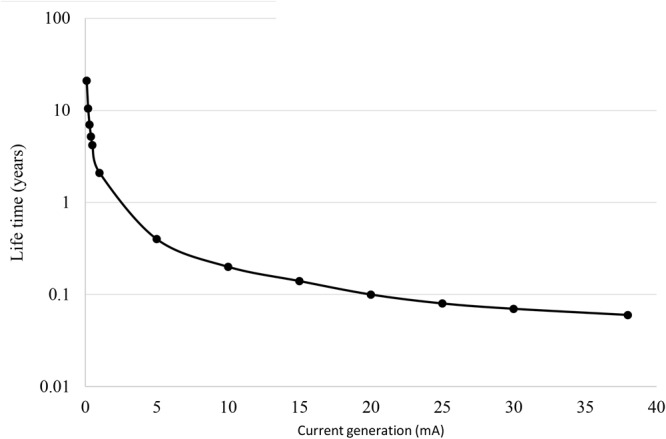
Estimation of bio-battery lifetime based on current generation.

The recharging time of the bio-battery does depend on the fuel content and its usage. Assuming, a typical 2% TOC value as glucose as maximum available “fuel” to run an MFC filled with marine sediment, one can estimate the time after which the sediment has to be renewed. According to the TOC content and the extracted current of 0.1 mA in these studied BESs (26.3 mA/m^2^ land use area or 154 mA/m^3^ anode volume), the MFC would run for 21 years assuming a low 10% Coulombic efficiency. Of course also all used-material should hold this durability (Supplementary Table 1). Long term experiments should be done to further clarify the durability of the bio-battery and clarify if all fuel is used over time. In case the current would be enhanced the refill-time of the bio-battery would decrease significantly as show in Figure 3. By direct installment of the MFC within the marine sediment, the MFC can warrant a prolonged electricity generation. Evenly plants like *Spartina anglica* can be integrated which will provide additional fuel via rhizodeposition and other loss of organic parts (e.g., via littering). Under natural conditions such planted marine sediment MFC located within a climate chamber has an estimated theoretical output between 0.14 and 0.34 W/m^2^ depending on the plant growth (Wetser et al., 2015a).

### Activated Carbon Granules Have Capacitive Behavior Which Allows External Electricity Storage in Microbial Medium Electrolytes

**FIGURE 4:**
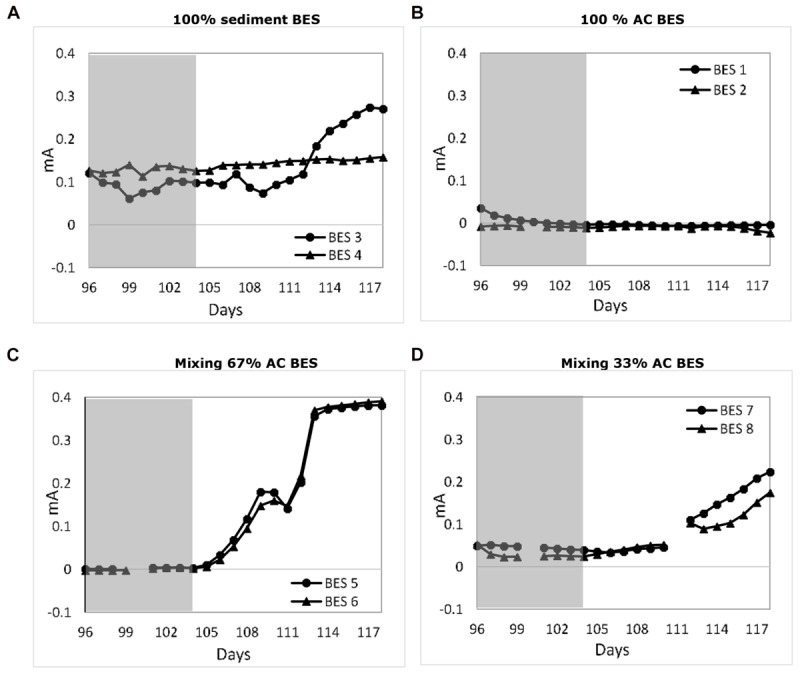
Effect of adding acetate on **2** week average daily performance at 1 k**Ω** on difference BES systems: **(A)** 100% sediment BES, **(B)** 100% AC BES, **(C)** mixing 67% AC BES, and **(D)** mixing 33% AC BES. Shaded area: Before adding acetate.

Activated carbon based packed granular electrodes are suited as electrode material in both bioanodes as well as biocathodes (Kalathil et al., 2011; Wei et al., 2011; Liu et al., 2014). Activated carbon is also used in supercapacitors to store electricity (Sevilla and Mokaya, 2014). However, to our best knowledge the role of these electrodes materials in saline microbial medium electrolytes is not investigated. The experimental result shows that the BESs with a 100% AC based anode (BES 1 and 2) are not able to generate electricity with just inoculum and no electron donor supply. This result can be clearly seen during long term operation with an external load (day 35–44) of BES 1 and 2 (Figure 2B). Even after adding 2 g/L acetate on day 105, these BESs did not producing any current (Figure 4B); possibly due to decay of the earlier supplied inoculum or absorption of nutrients on the AC may have limited the current generation. As such, these BESs acted as control experiments to validate the role of AC as electric charge storage material. During potentiostatic control (day 5–14 and day 44–55) the envisioned anode was actually acting as a cathode and electrical energy was added to the system at a controlled electrode potential of -100 mV (vs. Ag/AgCl). When the control mode was switched from potentiostatic control to external load (day 15–44 and day 56–72), BES 1 and 2 did generate a spontaneous anodic current starting at an electrode potential of 480 mV. In the later period, the current dropped harmonically toward zero until the electrode potential reached 14 mV. This phenomenon was evaluated as a kind of long-time charging (up to 11 days) and discharging (up to 29 days) behavior. The result shows that AC granules within anolyte medium are chargeable using externally supplied energy (i.e., electrical power) of which electricity was recovered later on. We assume that the inoculated electrochemically active bacteria did not play a crucial role during the charging and discharging while no electron donor was supplied. Still, microorganisms can do have capacitive properties which may affect the charge/discharge behavior (Schrott et al., 2011). The AC acted seemingly as a double layer chargeable capacitive electrode within a microbial growth medium electrolyte. This showed that bidirectional electron transfer was occurring within these systems. The nature of this electron transfer is in both directions (possible both bio- and electrochemically. The further mechanisms responsible for this need further clarification.

For BES 1 and 2, respectively, the charge recovery was 2.6 and 2.5% while the energy recovery was 0.29 and 0.26% resulting in an energy storage density of 22 kJ/m^3^ anode volume (0.02 Wh/kg used AC). This energy storage density is several order of magnitude lower than microbial rechargeable battery using acetate as the main energy carrier (Molenaar et al., 2016) or already optimized AC based supercapacitors (Sevilla and Mokaya, 2014). During discharge an oxygen reducing cathode was used consisting of graphite felt. The same electrode was used for a likely water oxidation reaction during the charging period. This graphite electrode was not specifically designed for both processes. The overall efficiencies are still low and need further investigation and optimization, for instance on counter electrode and redox couple, to maximize the storage capacity.

### Combining Marine Sediment With Activated Carbon (AC) Granules Generates *in situ* Electricity and Provides External Supplied Electricity Storage in a Bio Electrochemical System (BES)

Bioelectrochemical systems (BES 5, 6, 7, and 8) were also operated while combining marine sediment with activated carbon. After 156 days of operation, the marine sediment with AC BESs proved the capability to generate electricity and storage of externally supplied electricity (Figure 2C,D, 4C,D). On the long term performance (until day 105) without adding additional substrates (acetate), the BESs current generation dependent on the sediment fraction. Supplementary Table 3 provides the average performance after long term MFC operation of the various BESs. Adding sufficient AC was beneficial to increase electricity generation. The highest current and power density were obtained with BES 5 and 6 which contained 67% AC and 33% sediment. The BES 7 and 8 with a lower AC content of 33% resulted in a reduced electricity generation with 50% at a range comparable with BES 1 and 2 which contained only sediment. Apparent sufficient AC must be added to create a benefit of the material; this finding is in line with earlier work that also showed that 67% of granular electrode material applied is soil MFCs was most beneficial (Arends et al., 2012). The BESs were also temporarily poised at -100 mV (similar to the BES with 100% activated carbon) to store external supplied electricity. A similar long charging/discharging phenomena as compared to the 100% activated carbon (Figure 2B) was observed with 67% AC based BESs (Figure 2C) and 33% AC based BESs (Figure 2D) but was not shown with the 100% sediment anode BESs (Figure 2A). This result supports that activated carbon still had its capacitive chargeable behavior once mixed with marine sediment. The electricity could be stored for a long term period (10–20 days) while discharging took the same period. The observed phenomenon could possibly be exploited *in situ*, within marine sediments mixed with activated carbon based BES, allowing e.g., (intermittently produced) electricity storage. Once the observed charge/discharge effect is combined with an electrotrophic and electrogenic biofilm (operating at a sufficient low voltage range) (Yates et al., 2017); additional current could be also stored in microbial metabolites like CO or even acetate (Molenaar et al., 2016).

For a better understanding, the explanation of storage capacity phenomena will be further discussed for the period between day 44 and 72. During the potentiostatic control (day 44–55), anode voltage was kept at -100 mV because theoretically a more positive anode potential will help bacteria to gain more energy per electron transfer than a lower one (Wagner et al., 2010). As can be seen, during potentiostatic control only the 100% sediment BESs were able to generate electricity (see Figure 2A). While for the other BESs which did not generate current, their anode was receiving and storing electrons driven by the potentiostat (Supplementary Figures 1, 2). From Figure 2 one can see the more AC carbon fraction in the anode, the more negative i.e., cathodic current generation was observed. On day 56 when the control mode was switched to external load (MFC mode), stored electrons were released.

Current generation for 100% AC based BESs (1 and 2) increased from about -1.7 to 0.28 mA and for 67% AC based BESs (5 and 6) increased from about -1 mA to 0.29 mA while for 33% AC based BESs (7 and 8) increased from -0.05 to 0.1 mA and from 0.01 to 0.05 mA, respectively. This is supports that the storing capacity has a positive correlation with the amount of AC in the anode. After 16 days operation with external load (1000 Ω), on day 72 the current of all AC BESs decreased toward 0 mA, reaching a complete discharge. Similar phenomena were also observed during day 15–45.

Furthermore, acetate addition to the all sediment containing BESs enhanced current generation except for 100% AC based BESs (Figure 4, day 105). The sole AC BESs was producing zero current before and after acetate addition. For the sediment containing BESs, this enhancement indicates a substrate limitation (concentration or availability) in which the electrochemically active bacteria in the anode cannot utilize more complex remaining substrates from the sea sediment because of different microbial metabolism (Schroder, 2007). Added acetate is possibly utilized by the EAB which enhances their growth resulted in increasing current generation. Result of this research also indicated that 67% AC based BESs perform better compared to other BESs in this research. However, it remains unclear why the duplicate of only sea-sediment BES behaved differently upon the acetate addition.

### Internal Generated Electricity Storage Is Feasible in AC Granules Mixed With Marine Sediment BES

Capacitive bioanode electrodes can store internally generated electrons (obtained from the supplied fuel) within the double layer and/or capacitive biofilm of the bioelectrode (Deeke et al., 2012). The experiments, as explained before in a long term operation, showed that externally supplied electrical energy can apparently be stored in AC granules mixed with sea-sediment BESs. Considering this capability, further experiments were conducted to understand effect of AC granules marine sediment mixture on electricity storage from the internal source (i.e., the sediment itself). Experiments were conducted within two periods. The first period was before adding acetate (between day 72 and day 96) and the second one was after adding acetate (between day 118 and day 156). In these sets of experiments, internal charging was executed by setting the BESs at open cell voltage condition. Discharging was performed at constant controlled anode potential (65 mV vs. Ag/AgCl). Various charging times (CT) and discharging times (DT) were applied to identify feasible conditions for internal electricity supply and storage. The overall results did show that internal charging is feasible; although the phenomenon was depending on the type of electrode and composition of sediment and AC as further discussed.

**FIGURE 5:**
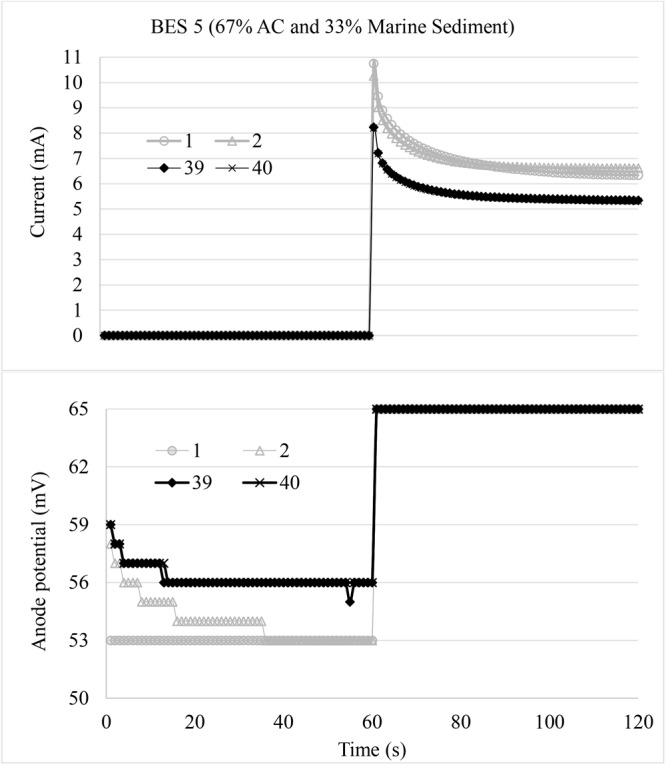
First cycle phenomena at CT 60 s and DT 60 s on day 142. The first two cycles and the last two cycles of experimental results were shown.

Figure 5 shows a typical response of storage behavior. Here the first cycle had an OCV of 4 h before the experiment was started and a DT of 120 s, which was followed by 39 charge and discharge cycles of respectively, 60 and 60 s. During discharge the average maximum current of the 40 cycle was 9 mA while the average stable current was 6 mA. During OCV the anode potential dropped repetitively with 10 mV. Based on 40 cycles experiment, the average stored charge on last 10 cycles was determined at 19.3 mC. In some experiments (as shown in Figure 5), the first cycle had a higher current than the consequent repetitive cycles; this was in-line with a longer OCV time used for the first cycles. An observation linked to this was the apparently higher drop of anode potential. The anode potential during OCV drops over time. Theoretically the EAB will generate electrons which polarize the electrode to more reduced conditions by reducing redox compounds and/or by direct electrode reduction and consequent accumulation of electric charge in the double layer of the bioelectrode. Also possible pH/salt gradients which negatively affect the anode potential may start to disappear (Timmers et al., 2012). After connecting the electrical circuit, the electrons will be released during discharging process. During the first cycle phenomena, the OCV is providing more time to create reducing conditions and allow a higher current. After repetitive applied OCV, more stable conditions arise which allow a stable discharge phenomenon.

Various experiments show successful and unsuccessful charging behaviors. Table 2 provides the overview of all conducted experiments with all key properties (Table 2A, Highest current; 2B, Stable current; 2C, Anode potential drop; and 2D, Stored charge). In the first phase experiment, BES 8 (e.g., Exp. 1c; 33% AC – 67% sediment) was the first marine AC based bioanode which showed a self-charging and storage capability. The stored charge from the applied CT of 180 s was 2.3 mC (Exp. 1c). All other BESs with AC within the bioanodes (BES 1, 2, 5, 6, and 7) were not able to discharge current (see all red in Tables 2A,B). Instead the BESs were externally charged by the potentiostat at 65 mV. This was shown by the negative (or zero) current both at the beginning of discharge period (average highest current on the last 10 cycles) and at the last period of discharge (average stable current on last 10 cycles). Thus unsuccessful self-charging in the first experiment could be caused by the low current generation as can be seen from day 72 when the first period was started, which was only 0.02 mA, from the anode of 67 and 33% AC BESs (Figure 2C,D). Such possible relatively short self-charging period (10–180 s), could be insufficient to (fully) charge a high capacitive AC anode (Sevilla and Mokaya, 2014).

**Table 2:** Internal generated electricity storage experiment.

Exp no.	CT (Sec)	DT (sec)	Cycles	Exp Day	BES 1	BES 2	BES 3	BES 4	BES 5	BES 6	BES 7	BES 8	BES 1	BES 2	BES 3	BES 4	BES 5	BES 6	BES 7	BES 8
					**A. Highest current as average from the**								**B. Stable current as average from the**							
					**final 10 cycles (mA)^∗^**								**final 10 cycles (mA)^∗^**							

1a	60	3780	40	75	–0.4	–0.5	0.1	0.1	–0.2	–0.3	0	0	–0.4	–0.5	0.1	0.1	–0.2	–0.3	0	0.03
1b	60	3780	40	78	–1.0	–1.1	0.9	1.1	–0.7	0.6	0	0.1	–0.7	–0.6	0.1	0.1	–0.2	0.2	0	0.02
1c	180	3780	40	83	–2.3	–1.1	1.8	2.2	–1.2	–1.4	0	0.1	–1.8	–0.6	0.1	0.1	–0.4	–0.6	0	0.03
1d	10	3780	40	90	–3.1	–1.2	0.3	0.3	–0.6	–1.3	0	0	–2.5	–0.7	0.1	0.1	–0.2	–0.6	0	0.02
2a	10	3780	40	127	n.a	n.a	2.3	1.3	8.0	7.5	1.5	0.9	n.a	n.a	0.7	0.4	5.8	5.8	0.9	0.6
2b	60	3780	40	133	n.a	n.a	5.9	3.4	4.2	3.9	1.7	1.4	n.a	n.a	0.7	0.4	2.6	2.4	0.0	0.6
2c	10	180	40	138	–6.3	–8.4	3.5	1.8	39	27	9.2	1.9	–6.3	–7.2	1.0	0.5	28	23	6.3	1.2
2d	60	180	40	138	–3.3	–9.5	9.0	4.4	39	42	6	2.4	–7.3	–5.4	1.0	0.5	22	38	3.1	1.0
2e	60	180	40	141	–0.6	–3.3	7.7	3.4	7.3	4.5	2.0	1.7	–0.4	–2.5	0.9	0.4	4.6	3.4	1.0	0.7
2f	3600	3600	10	141	2.3	0.0	18.0	22.1	9.2	3.9	6.2	11	1.8	0.2	1.0	0.4	3.7	2.6	1.0	0.6
2g	60	60	40	142	1.7	0.6	9.6	4.3	8.4	3.7	4.5	2.9	1.6	0.4	1.2	0.5	5.4	2.8	2.3	1.3
2h	10	10	40	142	1.1	0.7	4.2	1.8	7.7	3.5	4.0	2.6	1.8	0.5	1.4	0.6	5.8	2.9	2.7	1.7
2i	3600	10800	15	142	1.3	0.3	17.2	n.a	n.a	0.9	n.a	8.0	1.3	0.1	0.8	n.a	n.a	0.5	n.a	0.4
2j	3600	3600	15	142	n.a	n.a	n.a	22.7	4.9	n.a	5.8	n.a	n.a	n.a	n.a	0.4	2.0	n.a	1.0	n.a

					**C. Anode potential drop at open circuit from the**								**D. Average stored charge from the**							
					**discharge voltage from the final 10 cycles (mV)^∗∗^**								**finals 10 cycle (mC)^∗∗∗^**							

1a	60	3780	40	75	–1	–1	221	125	–1	–2	–1	2	n.c	n.c	1	0.9	n.c	n.c	n.c	0.8
1b	60	3780	40	78	–1	–2	126	68	1	17	0	1	n.c	n.c	5	4.7	n.c	n.c	n.c	0.5
1c	180	3780	40	83	–2	–1	188	99	–2	–7	0	1	n.c	n.c	13	14	n.c	n.c	n.c	2.3
1d	10	3780	40	90	–3	–1	131	88	–4	–16	–1	1	n.c	n.c	0.6	0.9	n.c	n.c	n.c	–0.1
2a	10	3780	40	127	n.a	n.a	251	156	39	73	18	27	n.a	n.a	8.2	3.9	151	348	7.3	13
2b	60	3780	40	133	n.a	n.a	273	186	13	62	21	42	n.a	n.a	34	21	112	68	45	35
2c	10	180	40	138	–18	–38	576	531	171	177	486	509	19.0	–97	7.1	4.2	117	36	33	6.8
2d	60	180	40	138	–8	–21	539	488	130	152	229	396	–26	–152	34	19	275	129	39	23
2e	60	180	40	141	–1	–4	315	206	9	8	18	44	–1.3	–6.7	30	14	23	14	13	15
2f	3600	3600	10	141	0	–3	483	443	16	11	59	240	483	–119	893	545	2383	657	1531	1600
2g	60	60	40	142	1	0	502	470	12	7	75	303	57	0.05	35	15	19	7.7	16	15
2h	10	10	40	142	1	0	446	357	11	7	43	89	5.2	0.3	5.4	2.4	3.8	1.1	2.5	1.8
2i	3600	10800	15	142	2	0	492	n.a	n.a	7	n.a	191	4085	4.7	785	n.a	n.a	616	n.a	1406
2j	3600	3600	15	142	n.a	n.a	n.a	442	12	n.a	64	n.a	n.a	n.a	n.a	539	1190	n.a	1389	n.a

*DT, Discharging Time; CT, Self Charging Time; n.a, not available; n.c, not calculated. First phase (non shaded): Before adding acetate; Second phase (shaded): After adding acetate; ^∗^ a positive current means that charge was recovered; a negative current shows that charge was supplied and not recovered; ^∗∗^ a negative value means that anode potential increased, while the positive values means that the anode potential decreased; ^∗∗∗^ a positive value means that self-charging from the anode worked while a negative value does mean that no charge was recovered due to negative or zero current. BES 1 and 2 = 100 % Activated Carbon (AC). BES 3 and 4 = 100 % Marine Sediment (MS). BES 5 and 6 = 67 % AC and 33% MS. BES 7 and 8 = 33% AC and 67% MS.*

Internal charging with AC marine bioanode was feasible with well performing bioanodes. With the insight of the first internal charging experiment, we executed second round of experiments after acetate addition; allowing further bioanode development and availability of more electrons for self-charging. These experiments were started after the BESs showed current. The AC mixed with sea-sediment BESs (0.17–0.35 mA) and also non-mixed sea-sediment BESs (0.16–0.27 mA) were generating the highest current (Figure 4).

After the addition of acetate and start-up of the bioanodes, the second period of self-charging experiments was conducted. Self-charging and storage was evidently shown with 33 and 67% AC bioanodes as well as the 100% marine BESs. The charge storage capacities were all positive for these electrodes as shown by the available data of the average stored charge in shaded area from Table 2D (Exps. 2a until 2j). For the 100% AC bioanode (BES 1 and 2), self-charge and storage was most evidently observed at the end of the experiments on day 142 (e.g., Exp. 2i) which was probably related due to the later start-up. During the OCV period of self-charging, the anode potential will typically drop because of EABs activity and charging of the double layer. The speed of anode potential drop is influenced by the capacitive properties of the anode material (Deeke et al., 2012). The more capacitive the anode, the slower the anode potential drop will be as more charge can be stored at a specific energy level (i.e., potential). Therefore for a non-capacitive electrode, the anode potential drop will faster approach the average i.e., stable open cell anode potential. This theory was in line with the presented experimental results in Table 2C for e.g., day 142. The anode potential drop was for the same CT time, the highest with 100% sediment BESs (3 and 4) and dropped with increasing AC content from 33% AC BESs (7 and 8), 67% AC BESs (5 and 6) to 100% AC BESs (1 and 2).

The successful self-charging and storage experiments support that chemical energy from the marine sediment and/or acetate was utilized with the EAB and used for self-charging of capacitive activated carbon bioanode during open circuit. This result is in line with single granule AC bioanode performance which showed electricity storage (Borsje et al., 2016). In this experiment we also found out that the sediment BESs were also able to store electricity (see e.g., Table 2, Exp. 2h). Evidently, this kind of self-charge and storage capability behavior could not be due to activated carbon since this material was absent. This could indicate other storage mechanisms in the sediment. One possible mechanism is that in the marine sediment easily accessible redox compounds exist which can take-up released electrons during self-charging time and releases them during discharging time. For instance during the self-charging, the released electrons from EAB can reduce NAD^+^ into NADH (Logan, 2009). When discharged at high anodic potential (65 mV), NADH can be oxidized back to NAD^+^ and release electrons. Another possible mechanism is the existence of sulfate reducing bacteria (SRB) and sulfide oxidizing bacteria (SOB). SRB are able to reduce sulfate into sulfide or elemental sulfur and later on the SOB can oxidize sulfide and elemental sulfur back to sulfate (Lee et al., 2014; Rao et al., 2016). Both species are naturally present in marine sediment and known to be able to have syntrophic growth during oxygen limitation (van den Ende et al., 1997). Sulfide oxidation was also proven to generate electricity in both a mixed culture (Rabaey et al., 2006) and a pure culture (Zhang et al., 2014) MFC system with the electrode as an electron acceptor. So, the metabolism of the SRB and SOB could play a role in delivering and taking up electrons while storing them via intracellular storage compounds. Naturally present humic substances in the marine sediment, such as humic acid and fluvic acid, could also involve in the charge storage (Tan et al., 2011). The humic substances could serve as electron shuttles in the marine sediment BES. Microorganism could transfer the electrons to the humic substances, then the reduces humic substances can rapidly reduces iron (III) oxides (Roden et al., 2010). The self-charge and storage capability could also be quinone based compounds (ter Heijne et al., 2018). The anode potential drop was much higher with pure marine sediment bioanodes (more than 300 mV) than the AC based electrode. This phenomenon shows the absence of activated carbon double layer capacitance as can be seen from experiment 2 h on Table 2C. We can expect that during the applied OCV the anode condition change. Earlier work on the electrochemicals characterization of comparable BESs (Plant-MFCs) explained that anode and membrane resistance decrease during current interruption (Timmers et al., 2012). As such, a lower internal resistance due to an OCV would lead to a temporarily higher current as observed with the self-charging experiment. To what extend the enhanced current was due to internal resistance changes and/or a potential sediment capacitive or other biological storage mechanisms was not assessed.

**FIGURE 6:**
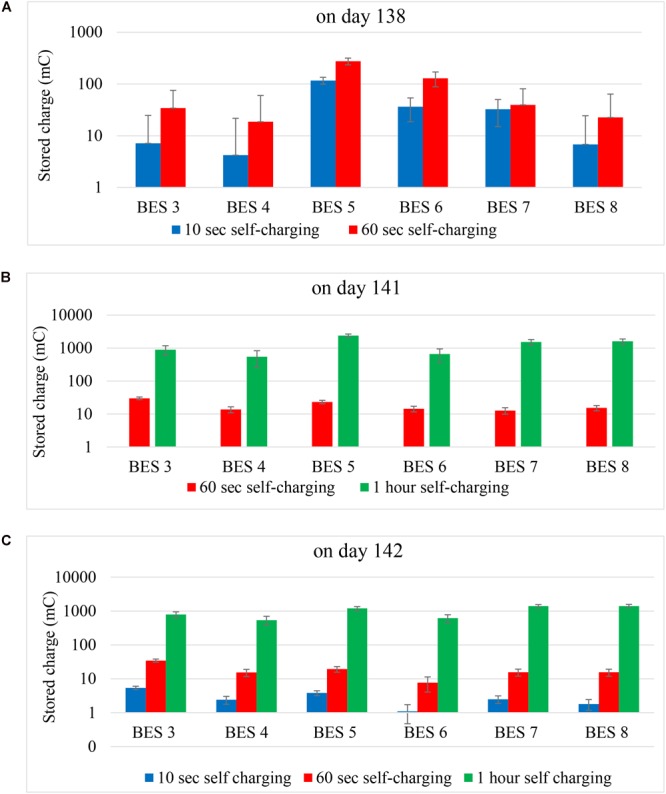
The effect of charging time on stored charge (mC) at different day: **(A)** on day 138, **(B)** on day 141, and **(C)** on day 142.

During the second phase of self-charging experiments, the effect of self-charging time was investigated by conducting electricity storage experiment on three different self-charging times (10 s, 60 s and 1 h). The experiment was conducted on day 138, 141, and 142. On day 138, 10 s and 60 s self-charging times with same discharging time (180 s) were performed. On day 141, 60 s and 1 h self-charging time with 180 s and 1 h discharging times, respectively, were carried out. Finally on day 142, 10 s, 60 s and 1 h self-charging times were executed with 10 s, 60 s and 3 h discharging time, respectively. In the self-charging experiments, stored electric charges must be generated by the bio anode of BES. We noticed that storage properties were not constant over time although the same CT and DT were applied (e.g., Table 2D, Exp 2d,e). Here the comparison of the results of the self-charging effect on stored charge was presented on the basis of each day of the experiment (Figure 6). BES 1 and 2 were excluded from discussion because they were not fully started up yet. The BES 1 and BES 2 started to generate current on day 141 right after the long charging-discharging period (Table 2B, Exp. 2f). This current generation was considered due to a long charging effect from external sources as described earlier in the Section “Activated Carbon Granules Have Capacitive Behavior Which Allows External Electricity Storage in Microbial Medium Electrolytes.”

A long self-charging time up to 1 h enhances charge storage. Based on results on Figure 6 one can see that for each BES the self-charging time has a positive correlation with stored charge. Stored charge increased when self-charging time was prolonged. Changing self-charging time from 10 to 60 s (Figure 6A,C) did increase stored charge between 1.2 and 9 times. Increasing the self-charging time with 60 times (from 60 s to 1 h; Figure 6B,C) increased stored charge between 22 and 40 times for the marine BESs 3 and 4, and 45 to 120 times for the AC marine bioanodes of BESs 5, 6, 7, and 8. The increase of stored charge for the AC bioanode did increase with several orders of magnitude which illustrates that even more charge could be stored. The self-charging bioanode had a maximum measured storage capacity of 2,383 mC (Table 2, Exp. 2f; BES 5) which corresponds to a volumetric storage of 3,666 C/m^3^ anode which enables electrons release at an anode energy level of 65 mV vs. Ag/AgCl. If we take a hypothetical cell voltage of 0.2 V, this would represent an electrical energy density of 0.3 mWh/kg mixed anode which is about 33,000 times lower than a super capacitor which can store up to 10 Wh/kg (Roldán et al., 2011). Furthermore, it is remarkable that the marine sediment BES acts like a capacitor similar to the charge storage behaviors as AC based bioanodes with a maximum volumetric storage of 1,373 C/m^3^ anode at a charge recovery of 57%. Taking the same hypothetical cell voltage of 0.2 V, this charge storage represents a potential capacitive battery energy property of 0.05 mWh/kg marine sediment.

**FIGURE 7:**
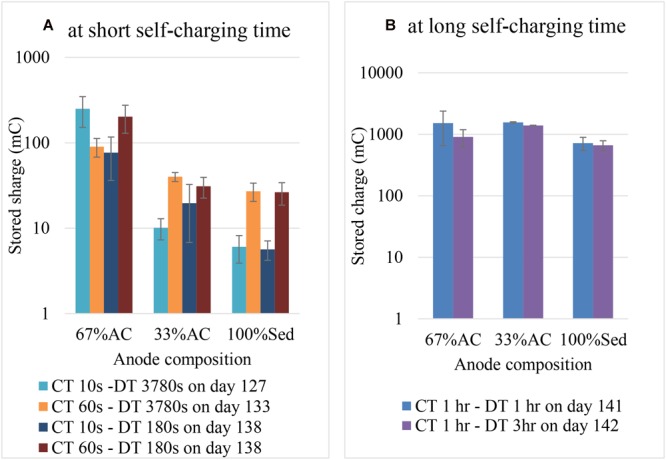
Effect of anode composition on stored charge at different charging and discharging time: **(A)** at short self-charging time, and **(B)** at long self-charging time.

At a short self-charging time, a higher percentage of AC enhanced stored charge. Based on an overview graph of all results (Supplementary Data) on the final 10 cycles on all electricity storage experiment, it can be seen that the number of measured charge was from the highest to the lowest as follows: 67% AC anode, 33% AC anode and 100% sediment (0% AC) anode. On the other hand, the effect of anode composition on stored charge is influenced by self-charging time. At short self-charging time (10 and 60 s) the effect of anode composition on stored charge is following the pattern of measured charge (Figure 7A). However at longer self-charging time (1 h) stored charge on different anode compositions are relatively the same (Figure 7B).

Figure 7 explains the influence of the anode composition on the stored charge. It shows the average stored charge from the duplicated BESs, namely 67% AC (BES 5 and 6), 33% AC (BES 7 and 8), and 100% Sed (BES 3 and 4). The storage properties of different anode compositions in Figure 7 should be compared according to the experimental day because the current output of each BES varied from one day to another day. Therefore, a comparison between self-charging time at 10 and 60 s should not be evaluated from day 127 and 133 but from day 138. Remarkable is that at shorter time more AC presences (67%) is enhancing the storage capability. On the other hand, at longer self-charging time the 100% marine sediment anode is seemingly able to store a similar amount of electric charge as much as in 67 and 33% AC anodes.

## Conclusion and Outlook

The study showed that marine sediment and activated carbon were able to store and generate electricity which is of possible use in rechargeable bio-batteries. The Dutch marine sediment was a suitable fuel to generate electricity. Installing this system within real outdoor sediments could warrant a long life-time due to the continuous supply of fuel. The used activated carbon granules showed within a microbial medium electrolyte a capacitive behavior which allowed external electricity storage. Combining marine sediment with activated carbon granules allowed both electricity generation from the supplied sediment and provided external supplied energy storage. The energy recovery of the bio-battery was rather low but can be optimized by an improved counter electrode. It was also shown that internal charging (during OCV) of bioanodes is feasible with mixed activated carbon and marine sediment. Evenly the marine sediment itself showed a similar storage behavior although the mechanisms responsible for this are to be further revealed. Charging time up to 1 h enhanced charge storage up 1,373 C/m^3^ with a charge recovery of 57% and an apparent capacitive battery energy property of 0.05 mWh/kg marine sediment.

## Author Contributions

ES designed and executed the experiments, calculated and wrote the first manuscript and revised the manuscript. DS was involved in designing the experiments, helped in the calculations and data analysis and revised the manuscript. CB did the final check and revision on the manuscript.

## Conflict of Interest Statement

The authors declare that the research was conducted in the absence of any commercial or financial relationships that could be construed as a potential conflict of interest.

## References

[B1] Abbas S. Z. Rafatullah M. Ismail N. Syakir M. I. (2017). A review on sediment microbial fuel cells as a new source of sustainable energy and heavy metal remediation: mechanisms and future prospective. *Int. J. Energy Res.* 41 1242–1264. 10.1002/er.3706

[B2] Arends J. B. A. Blondeel E. Tennison S. R. Boon N. Verstraete W. (2012). Suitability of granular carbon as an anode material for sediment microbial fuel cells. *J. Soils Sediments* 12 1197–1206. 10.1007/s11368-012-0537-6

[B3] Borsje C. Liu D. Sleutels T. H. J. A. Buisman C. J. N. ter Heijne A. (2016). Performance of single carbon granules as perspective for larger scale capacitive bioanodes. *J. Power Sources* 325 690–696. 10.1016/j.jpowsour.2016.06.092

[B4] de Haas H. Boer W. van Weering T. C. E. (1997). Recent sedimentation and organic carbon burial in a shelf sea: the North Sea. *Mar. Geol.* 144 131–146. 10.1016/S0025-3227(97)00082-0

[B5] Deeke A. Sleutels T. H. J. A. Hamelers H. V. M. Buisman C. J. N. (2012). Capacitive bioanodes enable renewable energy storage in microbial fuel cells. *Environ. Sci. Technol.* 46 3554–3560. 10.1021/es204126r 22332918

[B6] Dewan A. Beyenal H. Lewandowski Z. (2009). Intermittent energy harvesting improves the performance of microbial fuel cells. *Environ. Sci. Technol.* 43 4600–4605. 10.1021/es8037092 19603683

[B7] Helder M. Strik D. P. Hamelers H. V. Kuijken R. C. Buisman C. J. (2012). New plant-growth medium for increased power output of the plant-microbial fuel cell. *Bioresour. Technol.* 104 417–423. 10.1016/j.biortech.2011.11.005 22133604

[B8] Huggins T. Wang H. Kearns J. Jenkins P. Ren Z. J. (2014). Biochar as a sustainable electrode material for electricity production in microbial fuel cells. *Bioresour. Technol.* 157 114–119. 10.1016/j.biortech.2014.01.058 24534792

[B9] Jourdin L. Raes S. M. T. Buisman C. J. N. Strik D. P. (2018). Critical biofilm growth throughout unmodified carbon felts allows continuous bioelectrochemical chain elongation from CO2 up to caproate at high current density. *Front. Energy Res.* 6:7 10.3389/fenrg.2018.00007

[B10] Kalathil S. Lee J. Cho M. H. (2011). Granular activated carbon based microbial fuel cell for simultaneous decolorization of real dye wastewater and electricity generation. *New Biotechnol.* 29 32–37. 10.1016/j.nbt.2011.04.014 21718812

[B11] Kannan A. M. Renugopalakrishnan V. Filipek S. Li P. Audette G. F. Munukutla L. (2009). Bio-batteries and bio-fuel cells: leveraging on electronic charge transfer proteins. *J. Nanosci. Nanotechnol.* 9 1665–1678. 10.1166/jnn.2009.si03 19435024

[B12] Kim Y. Hatzell M. C. Hutchinson A. J. Logan B. E. (2011). Capturing power at higher voltages from arrays of microbial fuel cells without voltage reversal. *Energy Environ. Sci.* 4 4662–4667. 10.1039/C1EE02451E

[B13] Kumar G. G. Sarathi V. G. S. Nahm K. S. (2013). Recent advances and challenges in the anode architecture and their modifications for the applications of microbial fuel cells. *Biosens. Bioelectron.* 43 461–475. 10.1016/j.bios.2012.12.048 23452909

[B14] Lee D.-J. Liu X. Weng H.-L. (2014). Sulfate and organic carbon removal by microbial fuel cell with sulfate-reducing bacteria and sulfide-oxidising bacteria anodic biofilm. *Bioresour. Technol.* 156 14–19. 10.1016/j.biortech.2013.12.129 24480414

[B15] Liu J. Zhang F. He W. Zhang X. Feng Y. Logan B. E. (2014). Intermittent contact of fluidized anode particles containing exoelectrogenic biofilms for continuous power generation in microbial fuel cells. *J. Power Sources* 261 278–284. 10.1016/j.jpowsour.2014.03.071

[B16] Liu Y. Bond D. R. (2012). Long-distance electron transfer by *G. sulfurreducens* biofilms results in accumulation of reduced c-type cytochromes. *ChemSusChem* 5 1047–1053. 10.1002/cssc.201100734 22577055 PMC3500873

[B17] Logan B. E. (2009). Exoelectrogenic bacteria that power microbial fuel cells. *Nat. Rev. Microbiol.* 7:375. 10.1038/nrmicro2113 19330018

[B18] Logan B. E. Hamelers B. Rozendal R. Schröder U. Keller J. Freguia S. (2006). Microbial fuel cells: methodology and technology. *Environ. Sci. Technol.* 40 5181–5192. 10.1021/es0605016 16999087

[B19] Logan B. E. Regan J. M. (2006). Electricity-producing bacterial communities in microbial fuel cells. *Trends Microbiol.* 14 512–518. 10.1016/j.tim.2006.10.003 17049240

[B20] Lowy D. A. Tender L. M. Zeikus J. G. Park D. H. Lovley D. R. (2006). Harvesting energy from the marine sediment–water interface II: kinetic activity of anode materials. *Biosens. Bioelectron.* 21 2058–2063. 10.1016/j.bios.2006.01.033 16574400

[B21] Malvankar N. S. King G. M. Lovley D. R. (2014). Centimeter-long electron transport in marine sediments via conductive minerals. *ISME J.* 9:527. 10.1038/ismej.2014.131 25050525 PMC4303629

[B22] Malvankar N. S. Mester T. Tuominen M. T. Lovley D. R. (2012). Supercapacitors based on c-Type cytochromes using conductive nanostructured networks of living bacteria. *ChemPhysChem* 13 463–468. 10.1002/cphc.201100865 22253215

[B23] Manickam S. S. Karra U. Huang L. Bui N.-N. Li B. McCutcheon J. R. (2013). Activated carbon nanofiber anodes for microbial fuel cells. *Carbon* 53 19–28. 10.1016/j.carbon.2012.10.009

[B24] Mathuriya A. S. Yakhmi J. V. (2016). Microbial fuel cells – Applications for generation of electrical power and beyond. *Crit. Rev. Microbiol.* 42 127–143. 10.3109/1040841X.2014.905513 24903308

[B25] Middelburg J. J. Klaver G. Nieuwenhuize J. Wielemaker A. de Haas W. Vlug T. (1996). Organic matter mineralization in intertidal sediments along an estuarine gradient. *Mar. Ecol. Prog. Ser.* 132 157–168. 10.3354/meps132157

[B26] Molenaar S. D. Mol A. R. Sleutels T. H. J. A. ter Heijne A. Buisman C. J. N. (2016). Microbial rechargeable battery: energy storage and recovery through acetate. *Environ. Sci. Technol. Lett.* 3 144–149. 10.1021/acs.estlett.6b00051

[B27] Rabaey K. Van de Sompel K. Maignien L. Boon N. Aelterman P. Clauwaert P. (2006). Microbial fuel cells for sulfide removal. *Environ. Sci. Technol.* 40 5218–5224. 10.1021/es060382u 16999092

[B28] Rao A. M. F. Malkin S. Y. Hidalgo-Martinez S. Meysman F. J. R. (2016). The impact of electrogenic sulfide oxidation on elemental cycling and solute fluxes in coastal sediment. *Geochim. Cosmochim. Acta* 172 265–286. 10.1016/j.gca.2015.09.014

[B29] Reimers C. E. Tender L. M. Fertig S. Wang W. (2001). Harvesting energy from the marine sediment-water interface. *Environ. Sci. Technol.* 35 192–195. 10.1021/es001223s 11352010

[B30] Roden E. E. Kappler A. Bauer I. Jiang J. Paul A. Stoesser R. (2010). Extracellular electron transfer through microbial reduction of solid-phase humic substances. *Nat. Geosci.* 3 417–421. 10.1038/ngeo870

[B31] Roldán S. Granda M. Menéndez R. Santamaría R. Blanco C. (2011). Mechanisms of energy storage in carbon-based supercapacitors modified with a quinoid redox-active electrolyte. *J. Phys. Chem. C* 115 17606–17611. 10.1021/jp205100v

[B32] Sacco N. J. Figuerola E. L. M. Pataccini G. Bonetto M. C. Erijman L. Cortón E. (2012). Performance of planar and cylindrical carbon electrodes at sedimentary microbial fuel cells. *Adv. Biol. Waste Treat. Bioconv. Technol.* 126 328–335. 10.1016/j.biortech.2012.09.060 23142927

[B33] Schroder U. (2007). Anodic electron transfer mechanisms in microbial fuel cells and their energy efficiency. *Phys. Chem. Chem. Phys.* 9 2619–2629. 10.1039/B703627M 17627307

[B34] Schrott G. D. Bonanni P. S. Robuschi L. Esteve-Nuñez A. Busalmen J. P. (2011). Electrochemical insight into the mechanism of electron transport in biofilms of *Geobacter sulfurreducens*. *Electrochim. Acta* 56 10791–10795. 10.1016/j.electacta.2011.07.001

[B35] Scott K. Cotlarciuc I. Hall D. Lakeman J. B. Browning D. (2008). Power from marine sediment fuel cells: the influence of anode material. *J. Appl. Electrochem.* 38:1313 10.1007/s10800-008-9561-z

[B36] Seiter K. Hensen C. Schröter J. Zabel M. (2004). Organic carbon content in surface sediments—defining regional provinces. *Deep Sea Res. Part Oceanogr. Res. Pap.* 51 2001–2026. 10.1016/j.dsr.2004.06.014

[B37] Sevilla M. Mokaya R. (2014). Energy storage applications of activated carbons: supercapacitors and hydrogen storage. *Energy Environ. Sci.* 7 1250–1280. 10.1039/C3EE43525C

[B38] Tan W.-F. Norde W. Koopal L. K. (2011). Humic substance charge determination by titration with a flexible cationic polyelectrolyte. *Geochim. Cosmochim. Acta* 75 5749–5761. 10.1016/j.gca.2011.07.015

[B39] Tender L. M. Reimers C. E. Stecher H. A. III Holmes D. E. Bond D. R. Lowy D. A. (2002). Harnessing microbially generated power on the seafloor. *Nat. Biotechnol.* 20:821. 10.1038/nbt716 12091916

[B40] ter Heijne A. de Rink R. Liu D. Klok J. B. M. Buisman C. J. N. (2018). Bacteria as an electron shuttle for sulfide oxidation. *Environ. Sci. Technol. Lett.* 5 495–499. 10.1021/acs.estlett.8b00319 30135862 PMC6097799

[B41] Thomas Y. R. J. Picot M. Carer A. Berder O. Sentieys O. Barrière F. (2013). A single sediment-microbial fuel cell powering a wireless telecommunication system. *J. Power Sources* 241 703–708. 10.1016/j.jpowsour.2013.05.016

[B42] Timmers R. A. Strik D. P. Hamelers H. V. M. Buisman C. J. N. (2012). Characterization of the internal resistance of a plant microbial fuel cell. *Electrochim. Acta* 72 165–171. 10.1016/j.electacta.2012.04.023

[B43] Van de Broek M. Temmerman S. Merckx R. Govers G. (2016). Controls on soil organic carbon stocks in tidal marshes along an estuarine salinity gradient. *Biogeosciences* 13 6611–6624. 10.5194/bg-13-6611-2016

[B44] van den Ende F. P. Meier J. van Gemerden H. (1997). Syntrophic growth of sulfate-reducing bacteria and colorless sulfur bacteria during oxygen limitation. *FEMS Microbiol. Ecol.* 23 65–80. 10.1111/j.1574-6941.1997.tb00392.x

[B45] Wagner R. C. Call D. F. Logan B. E. (2010). Optimal set anode potentials vary in bioelectrochemical systems. *Environ. Sci. Technol.* 44 6036–6041. 10.1021/es101013e 20704197

[B46] Wei J. Liang P. Cao X. Huang X. (2011). Use of inexpensive semicoke and activated carbon as biocathode in microbial fuel cells. *Bioresour. Technol.* 102 10431–10435. 10.1016/j.biortech.2011.08.088 21924899

[B47] Wetser K. Liu J. Buisman C. Strik D. (2015a). Plant microbial fuel cell applied in wetlands: spatial, temporal and potential electricity generation of *Spartina anglica* salt marshes and *Phragmites australis* peat soils. *Biomass Bioenergy* 83 543–550. 10.1016/j.biombioe.2015.11.006

[B48] Wetser K. Sudirjo E. Buisman C. J. N. Strik D. P. (2015b). Electricity generation by a plant microbial fuel cell with an integrated oxygen reducing biocathode. *Appl. Energy* 137 151–157. 10.1016/j.apenergy.2014.10.006

[B49] Xie X. Criddle C. Cui Y. (2015). Design and fabrication of bioelectrodes for microbial bioelectrochemical systems. *Energy Environ. Sci.* 8 3418–3441. 10.1039/C5EE01862E

[B50] Yates M. D. Ma L. Sack J. Golden J. P. Strycharz-Glaven S. M. Yates S. R. (2017). Microbial electrochemical energy storage and recovery in a combined electrotrophic and electrogenic biofilm. *Environ. Sci. Technol. Lett.* 4 374–379. 10.1021/acs.estlett.7b00335

[B51] Zabihallahpoor A. Rahimnejad M. Talebnia F. (2015). Sediment microbial fuel cells as a new source of renewable and sustainable energy: present status and future prospects. *RSC Adv.* 5 94171–94183. 10.1039/C5RA15279H

[B52] Zhang L. L. Zhao X. S. (2009). Carbon-based materials as supercapacitor electrodes. *Chem. Soc. Rev.* 38 2520–2531. 10.1039/B813846J 19690733

[B53] Zhang T. Bain T. S. Barlett M. A. Dar S. A. Snoeyenbos-West O. L. Nevin K. P. (2014). Sulfur oxidation to sulfate coupled with electron transfer to electrodes by *Desulfuromonas* strain TZ1. *Microbiology* 160 123–129. 10.1099/mic.0.069930-0 24169815

