# Supplementary material for: Marine Sediment Mixed With Activated Carbon Allows Electricity Production and Storage From Internal and External Energy Sources: A New Rechargeable Bio-Battery With Bi-Directional Electron Transfer Properties
Source: Front Microbiol. 2019 May 14;10:934. doi: 10.3389/fmicb.2019.00934 (PMC6527962; doi:10.3389/fmicb.2019.00934)
Supplement: Supplementary file 1 [file Data_Sheet_1.docx]

Supplementary Material

Marine sediment mixed with activated carbon allows electricity production and storage from internal and external energy sources: a new rechargeable bio-battery with bi-directional electron transfer properties

Emilius Sudirjo^1, 2*^, Cees J.N Buisman^2^, David P.B.T.B Strik^2*^

^1^Government of Landak Regency, West Kalimantan, Indonesia

^2^Wageningen University & Research, Environmental Technology, Wageningen, The Netherlands

*** Correspondence:**David P.B.T.B Strik
[david.strik@wur.nl](mailto:david.strik@wur.nl)

Emilius Sudirjo
[emilius1.sudirjo@wur.nl](mailto:emilius1.sudirjo@wur.nl) / [emiliuss@gmail.com](mailto:emiliuss@gmail.com)

# Supplementary Data

Raw iviumstat data from charging and discharging experiment and its overview graph can be downloaded from <https://easy.dans.knaw.nl/ui/home> via this DOI link: <https://doi.org/10.17026/dans-xed-8qkv>

# Supplementary Figures

**Supplementary Figure 1.** Average daily performance of all bio electrochemical systems (BESs) for the entire research period. Figure 2 and Figure 4 in the article are scale-modified from this original figure.

**Supplementary Figure 2.**  Average daily performance of all bio electrochemical systems (BESs) for the entire research period. In this figure to show a clear result on positive current from all BESs, the y-axis is cut between -1 mA and 0.6 mA.

**Supplementary Figure 3.**  Acetate concentration in the anolyte of the BESs.

At day 150, all BESs were injected with 2 g/L NaAc. After sampling on day 117, additional 2 g/L NaAc was added into BES 1 and BES 2.

| 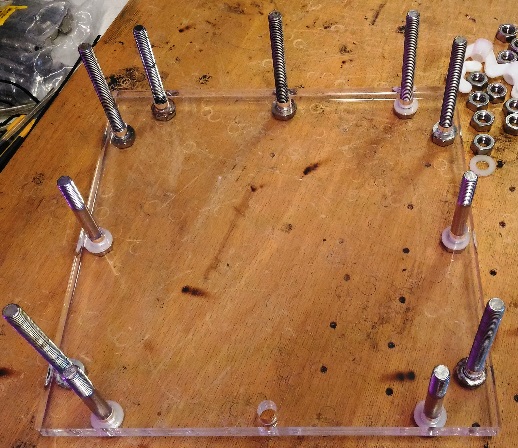   1. Solid end plate with bolts was prepared | 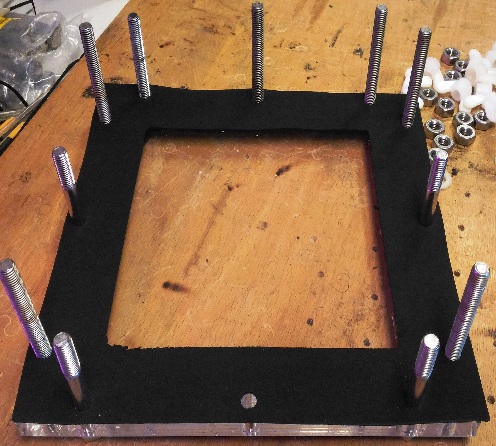   1. Black gasket was placed | | 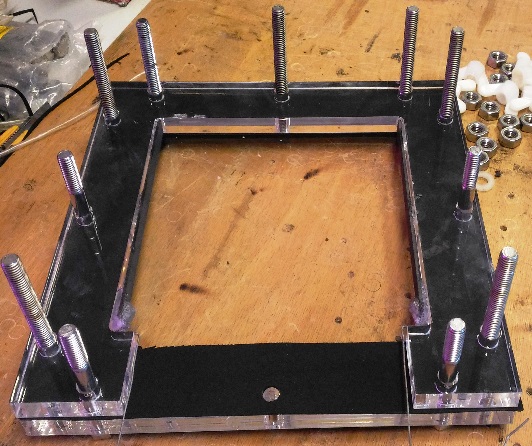   1. Anode plate was placed |
| --- | --- | --- | --- |
| 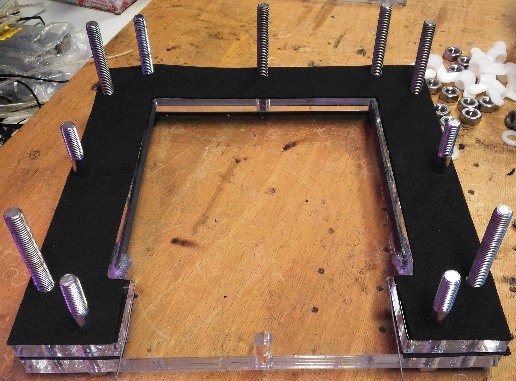   1. Black gasket was placed again | 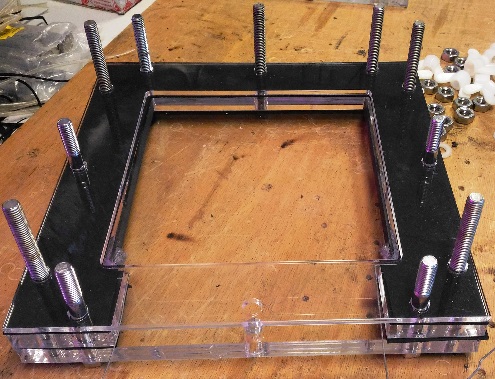   1. A support plate was placed | | 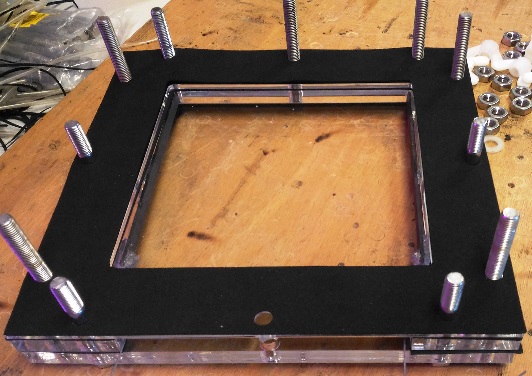   1. Another gasket was placed again |
| 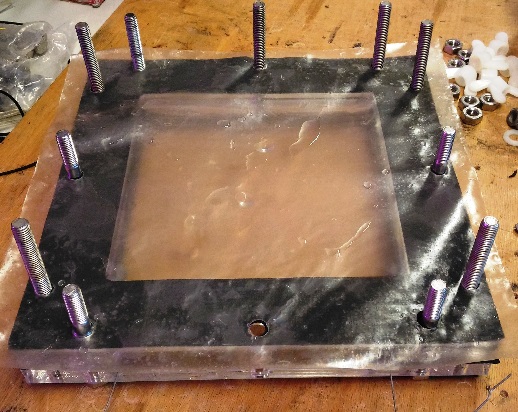   1. CEM was placed | 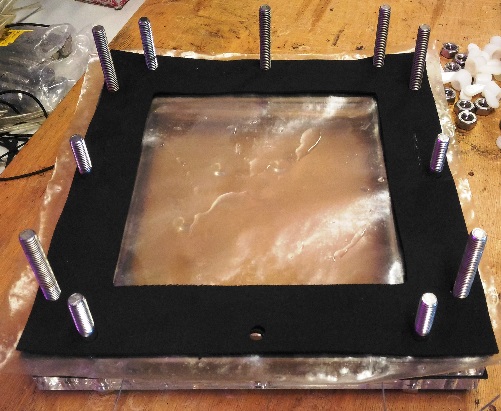   1. Gasket was placed again | | 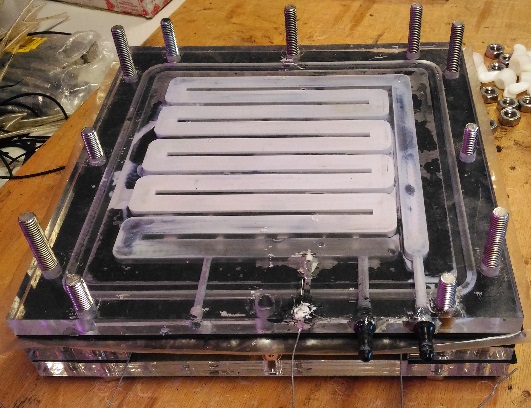   1. Finally, cathode plate was placed and reactor was tighten up with the nuts |
| 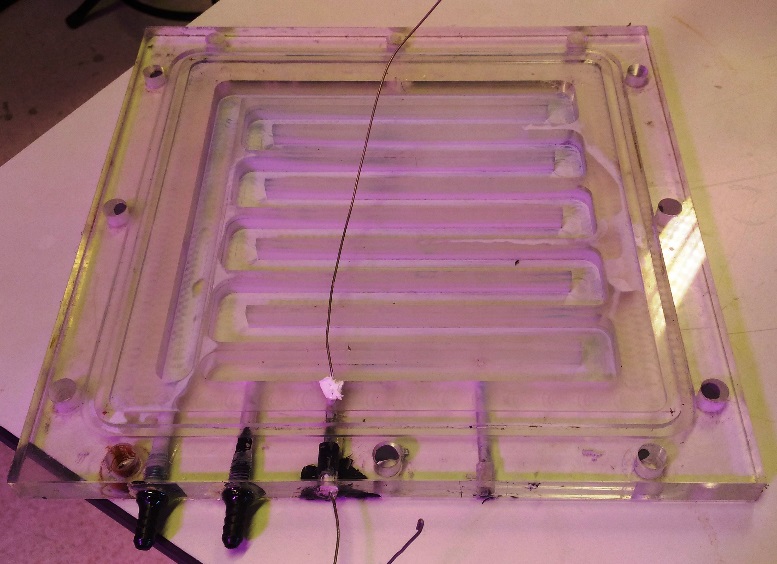   1. Cathode plate with winding channel | | 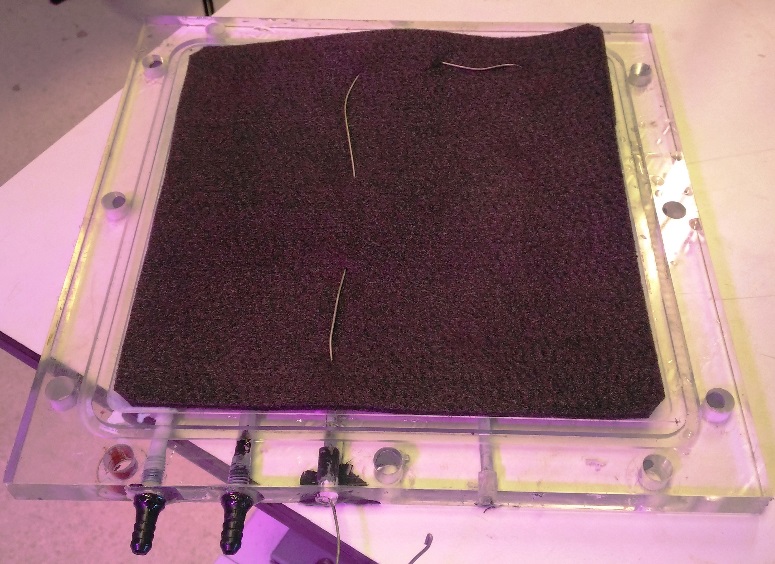   1. Graphite felt was woven with a current collector (Ti wire) | |

**Supplementary Figure 4.**  Stages of the BESs reactor preparation.

# Supplementary Tables

**Supplementary Table 1.**  Calculation for bio-battery life time using TOC value of marine sediment. Figure 4 in the article was made based on this calculation.

| TOC sea sediment is between 0.5-2% | |  |
| --- | --- | --- |
| Sediment weight in 100% sediment BES ( 3&4) system | 1027 | g |
| TOC weight in our BES 3& 4 (use 2% TOC for calculation) | 20.54 | g |
| TOC available based on 10% Coulombic efficiency | 2.054 | g |
| molar mass C | 12 | g/mol |
| mol C | 1.71167 | mol |
| number of C | 1.03E+24 | atom |
| number of electron^*^ | 4.12E+24 | atom |
| ^*^Based on acetate or glucose anodic oxidation reaction, 1 C generates 4 electrons (Heijne 2010) | | |
| measured electron flow (current) | **0.1** | mA |
| Q (C)=I(A).t(s) at 1 second | 0.0001 | C |
| Charge on 1 electron = 1.6 x 10^-19 Coulomb | |  |
| actual electron flow | 6.24E+14 | e/s |
| Time to finish the sediment fuel | 6.60E+08 | s **(20.9 years)** |
| Based on the above calculation, a simulation with other currents (up to 38mA which is the highest stable current as average from the final 10 cycles in this research) was made as following: | | |
| Current (mA) | Year |  |
| 0.2 | 10.5 |  |
| 0.3 | 7 |  |
| 0.4 | 5.2 |  |
| 0.5 | 4.2 |  |
| 1 | 2.1 |  |
| 5 | 0.4 |  |
| 10 | 0.2 |  |
| 15 | 0.14 |  |
| 20 | 0.1 |  |
| 25 | 0.08 |  |
| 30 | 0.07 |  |
| 38 | 0.06 |  |

TOC= Total Organic Carbon; C=Carbon

**Supplementary Table 2.**  Modified Hoagland Medium (Nitrate-less, sulphate-less, ammonium-bicarbonate-rich plant growth medium) composition used in this study.

| **Compounds** | **Concentration (mg/L)** |
| --- | --- |
| Macronutriens |  |
| NH_4_HCO_3_ | 553.43 |
| CaCl_2_ | 222 |
| NH_4_H_2_PO_4_ | 115.08 |
| MgSO_4_.7H_2_O | 123.24 |
| KCl | 223.68 |
| NaCl | 5000 |
| C_14_H_18_N_3_O_10_Fe | 10 |
| Na_2_SiO_3_.9H_2_O | 142.10 |
| Micronutrients (Arno-E) |  |
| KCl | 0.466 |
| H_3_BO_3_ | 0.193 |
| MnSO_4_.H_2_O | 0.042 |
| ZnSO_4_.7H_2_O | 0.072 |
| CuSO_4_.5H_2_O | 0.016 |
| H_2_MoO_4_ (85% MoO_3_) | 0.01 |
| NaFeDTPA (10% Fe) | 1.498 |

**Supplementary Table 3.**  Average performance of the BESs reactors at the MFC mode from day 117 to 118 before the second charging and discharging experiment was performed

| **Properties** | **BES 1** | **BES 2** | **BES 3** | **BES 4** | **BES 5** | **BES 6** | **BES 7** | **BES 8** |
| --- | --- | --- | --- | --- | --- | --- | --- | --- |
| Anode Potential (mV) | 95.91 | 104.93 | -403.28 | -303.29 | -426.38 | -438.56 | -11.45 | -269.42 |
| Cell Potential (mV) | -4.40 | -20.01 | 273.08 | 156.06 | 381.02 | 389.17 | 213.59 | 159.22 |
| Current (mA) | 0.00 | -0.02 | 0.27 | 0.16 | 0.38 | 0.39 | 0.21 | 0.16 |
| Current densities (mA/m2) | -1.16 | -5.27 | 71.86 | 41.07 | 100.27 | 102.41 | 56.21 | 41.90 |
| Current densities (mA/m3) | -6.76 | -30.79 | 420.12 | 240.09 | 586.18 | 598.72 | 328.60 | 244.96 |
| Power (mW) | n.a. | n.a. | 0.07 | 0.02 | 0.15 | 0.15 | 0.05 | 0.03 |
| Power densities (mW/m2) | n.a. | n.a. | 19.62 | 6.41 | 38.20 | 39.86 | 12.01 | 6.67 |
| Power densities (mW/m3) | 0.03 | 0.62 | 114.73 | 37.47 | 223.35 | 233.00 | 70.19 | 39.00 |
